# Early Post-Treatment Eosinophil Elevation and Survival Outcomes in Metastatic Renal Cell Carcinoma Treated with First-Line VEGFR-TKI Monotherapy: A Turkish Multicenter Retrospective Cohort Study

**DOI:** 10.3390/biomedicines14071621

**Published:** 2026-07-18

**Authors:** Oktay Halit Aktepe, Shamil Rustamov, Rezan Berkay Izgor, Osman Butun, Seren Karakaya, Tugce Ulasli, Suayib Yalcin

**Affiliations:** 1 Department of Medical Oncology, Dokuz Eylül University, Izmir 35330, Türkiye; 2 Department of Internal Medicine, Delta Hospital, Istanbul 34841, Türkiye; 3 Department of Internal Medicine, Faculty of Medicine, Dokuz Eylul University, Izmir 35330, Türkiye; 4 Department of Medical Oncology, Acibadem Izmir Kent Hospital, Izmir 35620, Türkiye; 5 Department of Medical Oncology, Hacettepe University Cancer Institute, Ankara 06230, Türkiye

**Keywords:** post-treatment eosinophil elevation, metastatic renal cell carcinoma, prognostic biomarker, survival outcomes, tyrosine kinase inhibitors

## Abstract

**Background/Objectives**: We aimed to evaluate the association between early post-treatment eosinophil (Eo) elevation and survival outcomes in metastatic renal cell carcinoma (mRCC) treated with vascular endothelial growth factor receptor tyrosine kinase inhibitors (VEGFR-TKIs). We also assessed whether early post-treatment Eo elevation provided prognostic information beyond the International Metastatic Renal Cell Carcinoma Database Consortium (IMDC) model. **Methods**: This Turkish retrospective multicenter cohort study included 280 patients with mRCC who received first-line VEGFR-TKI monotherapy between 2015 and 2025. Early post-treatment Eo elevation was defined as a post-baseline Eo percentage > 5% in the complete blood count obtained within 15 days before the first computed tomography assessment performed for response/progression evaluation. Progression-free survival (PFS) and overall survival (OS) were estimated using Kaplan–Meier methods and compared with log-rank tests. Cox regression models assessed independent prognostic associations. Time-dependent receiver operating characteristic (ROC) curve analyses were performed to evaluate the incremental prognostic value of early post-treatment Eo elevation beyond IMDC risk. **Results**: The median age was 61 years, and 205 patients (73.2%) were male. Early post-treatment Eo elevation was observed in 88 patients (31.4%). Median PFS was longer in patients with early post-treatment Eo elevation than in those without Eo elevation (15.1 vs. 10.1 months; *p* < 0.001). Median OS was also longer in the Eo elevation group (70.0 vs. 38.6 months; *p* < 0.001). In multivariable analysis, early post-treatment Eo elevation remained independently associated with improved PFS (hazard ratio [HR]: 0.60, 95% confidence interval [CI]: 0.44–0.81, *p* = 0.001) and OS (HR: 0.51, 95% CI: 0.33–0.80, *p* = 0.004). The combined IMDC plus Eo model showed higher time-dependent area under the curve values than the IMDC model alone. **Conclusions**: Early post-treatment Eo elevation was independently associated with improved PFS and OS, and the combined model showed greater prognostic discrimination than IMDC risk alone. Given that Eo status was assessed after treatment initiation, residual selection bias cannot be excluded, and prospective validation is required before clinical implementation.

## 1. Introduction

Kidney cancer ranked as the 14th most frequently diagnosed malignancy globally and the 15th leading cause of cancer-related mortality in 2020 [1]. Renal cell carcinoma (RCC) represents the predominant subtype of kidney cancer, comprising nearly 90% of diagnosed cases [2]. While localized RCC carries a favorable 5-year relative survival of 92.7%, this decreases dramatically to 15.3% in patients presenting with distant metastases [3], indicating poor prognosis. The treatment of metastatic RCC (mRCC) has shifted from vascular endothelial growth factor receptor (VEGFR)-targeted tyrosine kinase inhibitor (TKI) monotherapy to combination regimens with immune-checkpoint inhibitors (ICIs), which have shown superior survival in phase III trials [4,5,6]. However, many patients continue to receive VEGFR-TKIs alone because of access issues, comorbidities, or toxicity concerns, making the assessment of prognostic markers under TKI monotherapy clinically significant [7]. In this context, identifying simple and widely available prognostic biomarkers remains an unmet clinical need, particularly for patients who continue to be treated with VEGFR-TKI monotherapy.

The International Metastatic Renal Cell Carcinoma Database Consortium (IMDC) model is one of the most widely used prognostic classification systems in mRCC [8]. It stratifies patients into favorable-, intermediate-, and poor-risk groups using baseline clinical and laboratory parameters, including performance status, time from diagnosis to systemic treatment, hemoglobin, calcium, neutrophil count, and platelet count. Its main strengths are its simplicity, reproducibility, reliance on routinely available variables, and broad use in clinical trials and real-world practice. However, the IMDC model is based on pretreatment static variables and does not capture early treatment-period biological changes, treatment-related host responses, or dynamic peripheral blood markers. Therefore, biomarkers assessed during treatment may provide complementary prognostic information beyond baseline IMDC risk assessment.

Beyond the IMDC risk model, several clinical, radiological, and biochemical parameters have been investigated as prognostic markers in patients with mRCC treated with VEGFR-TKIs. These include histologic subtype, tumor grade, metastatic site distribution, sarcopenia and other body-composition parameters, inflammatory markers, and lipid profile [9,10,11,12,13,14]. However, most of these markers are baseline variables and may not fully reflect early treatment-related host response or evolving tumor biology. Therefore, simple post-treatment blood-based markers may provide complementary prognostic information beyond conventional risk models.

Peripheral blood eosinophil (Eo) level, as a routinely measured laboratory parameter, has recently attracted attention as a potential marker of treatment response and survival. However, its role in mRCC treated with VEGFR-TKI monotherapy has not been fully clarified. In the tumor microenvironment (TME), Eos can exert antitumor effects by secreting pro-inflammatory cytokines, releasing cytotoxic granules, and promoting T-cell recruitment [15]. Additionally, experimental studies have suggested that they participate in the modulation of angiogenesis and enhance responsiveness to ICIs [15,16]. However, eosinophilia has also been associated with immune-related adverse events during ICI therapy, underscoring its complex role in cancer biology [17]. There is little information available regarding the clinical significance of peripheral blood Eo counts in the context of RCC, especially in patients receiving targeted agents. Treatment results have been linked to early alterations in circulating blood markers, including hemoglobin level, platelet count, and neutrophil-to-lymphocyte ratio [18,19,20]. However, whether early post-treatment Eo elevation provides prognostic information in patients with mRCC receiving VEGFR-TKI therapy remains inadequately investigated. A study in mRCC patients receiving sorafenib reported an Eo percentage elevation in about one-third of patients (~36%), which was linked to better survival [21]. In this retrospective study, we evaluated whether early post-treatment Eo elevation before the first radiological response/progression assessment was independently associated with progression-free survival (PFS) and overall survival (OS) in patients with mRCC receiving first-line VEGFR-TKI monotherapy, beyond established clinicopathological prognostic factors.

## 2. Materials and Methods

### 2.1. Patient Population and Study Design

We conducted a retrospective cohort study including 280 patients with mRCC who received first-line VEGFR-TKI monotherapy between January 2015 and January 2025 at the participating oncology centers in Turkey. Patients were identified from institutional oncology databases and electronic medical records. Baseline demographic and clinicopathologic data included age, sex, histological subtype (clear cell vs. non-clear cell), tumor grade, and metastatic sites. Risk stratification was performed according to the IMDC criteria, classifying patients into favorable, intermediate, or poor risk groups [8]. Treatment histories, including the type of first-line VEGFR-TKIs and subsequent lines of systemic therapy (nivolumab, axitinib, everolimus, or cabozantinib), were collected. Eligibility criteria included: (1) age ≥ 18 years, (2) histologically confirmed mRCC, (3) available complete blood count and serum biochemistry data at baseline, (4) a follow-up complete blood count obtained within 15 days before the first computed tomography assessment performed for response/progression evaluation, and (5) treatment with VEGFR-TKIs, including sunitinib, pazopanib, or cabozantinib, as first-line therapy. Patients with both clear-cell and non–clear-cell histologies were eligible.

Patients who had received ICIs as first-line treatment, underwent nephrectomy after systemic therapy initiation, or had missing laboratory or follow-up data were excluded from the analysis. Additionally, patients with known conditions or medications that could influence peripheral Eo levels, including concurrent hematologic or autoimmune diseases, active parasitic or allergic disease at treatment initiation, and chronic corticosteroid or other immunosuppressive medication use, were excluded.

Peripheral blood Eo percentages were recorded at baseline and at the time of the first radiological response/progression assessment. The follow-up Eo value was defined as the complete blood count obtained within 15 days before the first computed tomography assessment performed for response/progression evaluation. In routine clinical practice, this first radiological assessment was generally scheduled within the first three months after initiation of VEGFR-TKI therapy. The 5% cut-off was based on Wang et al., who defined post-treatment Eo percentage escalation as Eo > 5% at 4–8 weeks after sorafenib initiation [21]. This cut-off was selected a priori as a literature-adapted threshold and was not derived from the present cohort by receiver operating characteristic (ROC) analysis or maximally selected rank statistics. In the present study, the same cut-off was applied to the pre-imaging complete blood count obtained within 15 days before the first radiological assessment, generally within the first three months of VEGFR-TKI therapy. Early post-treatment Eo elevation was therefore defined as an Eo percentage > 5% in this pre-imaging complete blood count. Based on this predefined cut-off, patients were categorized into post-treatment Eo elevation (>5%) and no post-treatment Eo elevation (≤5%) groups.

### 2.2. Statistical Analysis

Continuous variables were summarized as medians with interquartile ranges (IQR) or as means with standard deviations (SD), according to the distribution of the data. Categorical variables were reported as frequencies and percentages. Between-group comparisons were performed using the Mann–Whitney U test for continuous variables and the chi-square test or Fisher’s exact test for categorical variables, as appropriate. PFS was defined as the time from initiation of first-line VEGFR-TKI therapy to radiological progression or death from any cause, whichever occurred first. OS was defined as the time from initiation of first-line VEGFR-TKI therapy to death from any cause. Patients without an event were censored at the date of last follow-up. Survival distributions were estimated using the Kaplan–Meier method and compared with the log-rank test.

The associations between clinicopathologic variables and survival outcomes were evaluated using Cox proportional hazards regression models. Univariable Cox models were first constructed for candidate prognostic variables. Variables with a *p* value ≤ 0.20 in univariable analysis, together with clinically relevant covariates, were entered into multivariable Cox regression models. Results were expressed as hazard ratios (HR) with 95% confidence intervals (CI).

To address potential guarantee-time (immortal-time) bias arising from ascertainment of eosinophil status after treatment initiation, a landmark analysis was performed using a 3-month landmark corresponding to the first radiological assessment window. Patients with an event before the landmark were excluded, eosinophil status was fixed at the landmark, and survival was recalculated from the landmark. As the exact timing of the first assessment varied, we conducted sensitivity analyses at 2- and 4-month landmarks; consistency of the hazard ratios across these landmarks was taken to indicate robustness of the findings to the specific landmark chosen.

To assess whether the prognostic association of early post-treatment Eo elevation varied according to baseline IMDC risk, an interaction analysis was performed after dichotomizing IMDC risk into favorable/intermediate versus poor risk. An interaction term between early post-treatment Eo elevation and poor IMDC risk was included in the Cox proportional hazards model. The model was adjusted for first-line VEGFR-TKI type, histology, tumor grade, and clinically relevant metastatic sites, including lung, liver, bone, adrenal, and brain metastases. The incremental prognostic value of early post-treatment Eo elevation beyond the IMDC risk model was assessed using time-dependent ROC analysis for OS. Time-dependent area under the curve (AUC) values were calculated at 12 and 24 months for early post-treatment Eo elevation alone, IMDC risk alone, and the combined IMDC plus Eo elevation model.

All statistical tests were two-sided, and a *p* value < 0.05 was considered statistically significant. Descriptive statistics and conventional Cox regression analyses were performed using IBM SPSS Statistics version 27.0 (IBM Corp., Armonk, NY, USA). Kaplan–Meier figures, IMDC subgroup and interaction analyses, landmark analyses and time-dependent ROC analyses were performed using R software version 4.6.0 (R Foundation for Statistical Computing, Vienna, Austria).

## 3. Results

### 3.1. Baseline Characteristics and Treatment Patterns

A total of 280 patients with mRCC were included in the analysis. Patient characteristics of the overall cohort and according to early post-treatment Eo elevation status are presented in Table 1. The median age of the overall cohort was 61 years (IQR: 54–67), and *n* = 205 (73.2%) patients were male. Clear-cell histology was observed in *n* = 219 (78.2%) patients, and high-grade disease, defined as tumor grade III–IV, was present in *n* = 199 (71.1%). Lung metastasis was the most frequent metastatic site (*n* = 201, 71.8%), followed by bone (*n* = 73, 26.1%), liver (*n* = 59, 21.1%), adrenal gland (*n* = 24, 8.6%), and brain metastases (*n* = 18, 6.4%). According to the IMDC risk classification, *n* = 41 (14.6%) patients were categorized as favorable risk, *n* = 167 (59.6%) as intermediate risk, and *n* = 72 (25.7%) as poor risk.

When patients were stratified according to early post-treatment Eo elevation status, *n* = 192 (68.6%) were classified into the no Eo elevation group (≤5%) and *n* = 88 (31.4%) into the Eo elevation group (>5%). Baseline age was comparable between the two groups, with a median age of 60 years (IQR: 53–68) in the no Eo elevation group and 61 years (IQR: 56–66) in the Eo elevation group (*p* = 0.361). The distributions of sex, tumor grade, and the IMDC risk category were also similar between the groups. Clear-cell histology was more common in the Eo elevation group than in the no Eo elevation group (*n* = 75, 85.2% vs. *n* = 144, 75.0%), although the difference did not reach statistical significance (*p* = 0.054). Baseline Eo percentage was significantly higher in the Eo elevation group than in the no Eo elevation group (3.73 vs. 2.52, *p* < 0.001). Regarding metastatic sites, lung metastasis was more frequent in the Eo elevation group (*n* = 72, 81.8% vs. *n* = 129, 67.2%; *p* = 0.012), whereas bone metastasis was less frequent (*n* = 16, 18.2% vs. *n* = 57, 29.7%; *p* = 0.042). The frequencies of liver, adrenal, and brain metastases were comparable between patients with and without early post-treatment Eo elevation.

The mean baseline Eo percentage was 2.90% (SD: 1.00) in the overall cohort and was significantly higher in the Eo elevation group than in the no Eo elevation group (3.73% [SD: 0.64] vs. 2.52% [SD: 0.91], *p* < 0.001). The mean early post-treatment Eo percentage was 3.62% (SD: 1.51) in the overall cohort, 2.77% (SD: 0.93) in the no Eo elevation group, and 5.50% (SD: 0.45) in the Eo elevation group. Because early post-treatment Eo percentage was used to define the study groups, no between-group statistical comparison was performed for this variable.

Treatment patterns according to early post-treatment Eo elevation status (>5% vs. ≤5%) are summarized in Table 2. In the overall cohort, pazopanib was the most commonly used first-line VEGFR-TKI (53.2%), followed by sunitinib (36.1%) and cabozantinib (10.7%). The distribution of first-line treatment differed significantly between the Eo elevation and no Eo elevation groups (*p* < 0.001), with pazopanib being more frequently used among patients with Eo elevation. Among patients who received second-line therapy, nivolumab was the most frequently administered agent (54.5%), followed by axitinib (23%), everolimus (20.1%), and cabozantinib (2.4%). The distribution of second-line treatment did not significantly differ according to early post-treatment Eo elevation status (*p* = 0.399). In the third-line setting, axitinib was the most commonly used treatment (55%), followed by nivolumab (31.2%), everolimus (10%), and cabozantinib (3.8%), with no significant difference between groups (*p* = 0.550).

### 3.2. Survival Outcomes

**Figure 1:**
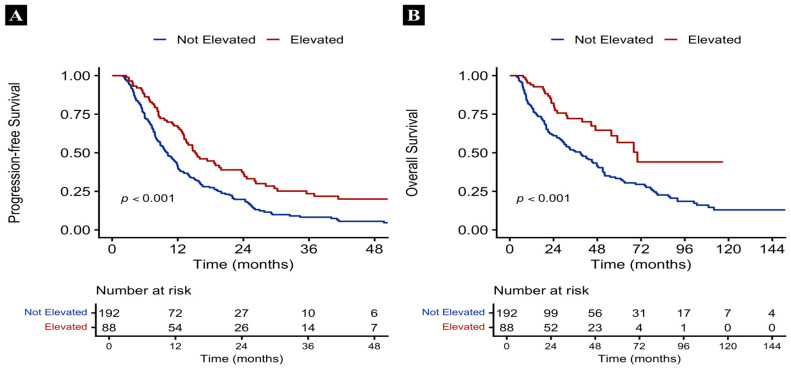
Kaplan–Meier curves depicting PFS (**A**) and OS (**B**) according to early post-treatment Eo elevation status (>5% vs. ≤5%) in patients with mRCC receiving first-line VEGFR-TKI monotherapy. Abbreviations: Eo: eosinophil; mRCC: metastatic renal cell carcinoma; OS: overall survival; PFS: progression-free survival; VEGFR-TKI: vascular endothelial growth factor receptor tyrosine kinase inhibitor.

During the follow-up period, a total of 233 progression events were recorded. The median PFS of the overall cohort was 11.8 months (95% CI: 10.3–13.4). Patients with early post-treatment Eo elevation had a longer median PFS than those without early post-treatment Eo elevation (15.1 months, 95% CI: 11.5–18.7 vs. 10.1 months, 95% CI: 8.4–11.8; *p* < 0.001; Figure 1A). A total of 157 deaths were observed during a median follow-up of 56.5 months. The median OS of the overall cohort was 47 months (95% CI: 39.6–54.3). As shown in Figure 1B, patients with early post-treatment Eo elevation had a markedly longer median OS than those without early post-treatment Eo elevation (70 months, 95% CI: 53.04–86.96 vs. 38.6 months, 95% CI: 28.5–48.7; *p* < 0.001).

**Figure 2:**
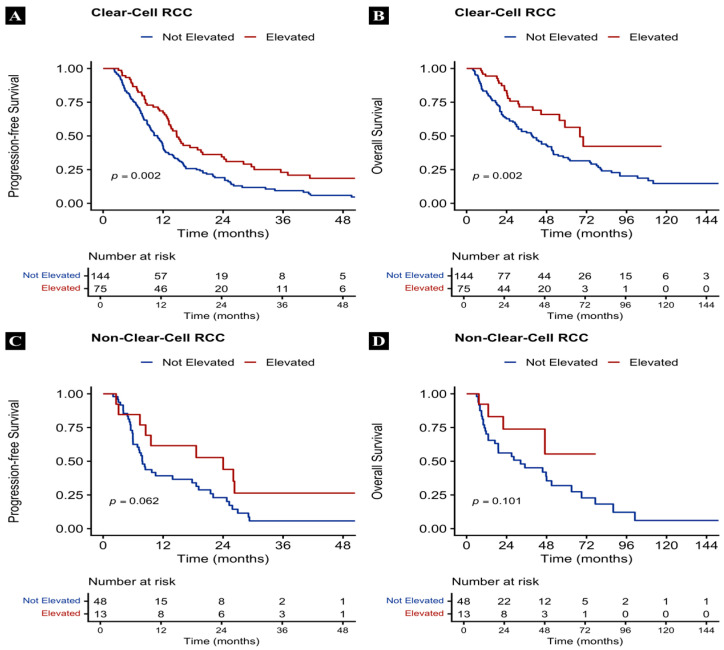
Kaplan–Meier curves depicting PFS and OS according to early post-treatment Eo elevation status (>5% vs. ≤5%) in clear-cell and non–clear-cell mRCC. Panels (**A**,**B**) show PFS and OS in clear-cell RCC, whereas panels (**C**,**D**) show PFS and OS in non–clear-cell RCC. Abbreviations: Eo: eosinophil; mRCC: metastatic renal cell carcinoma; OS: overall survival; PFS: progression-free survival; RCC: renal cell carcinoma.

Regarding survival outcomes according to histologic subtype, among patients with clear-cell RCC, those with early post-treatment Eo elevation demonstrated longer median PFS and OS than those without early post-treatment Eo elevation. Median PFS was 14.8 months (95% CI: 12.6–16.9) in patients with early post-treatment Eo elevation versus 10.9 months (95% CI: 9.1–12.6) in those without early post-treatment Eo elevation (*p* = 0.002; Figure 2A). Similarly, median OS was 68 months (95% CI: 52.7–83.3) in patients with early post-treatment Eo elevation compared with 39.4 months (95% CI: 28.4–50.3) in those without early post-treatment Eo elevation (*p* = 0.002; Figure 2B). In the non–clear-cell RCC subgroup, patients with early post-treatment Eo elevation also showed numerically longer PFS and OS than those without early post-treatment Eo elevation. Median PFS was 24.0 months (95% CI: 1.0–47.0) in patients with early post-treatment Eo elevation versus 7.8 months (95% CI: 6.5–9.1) in those without early post-treatment Eo elevation (*p* = 0.062; Figure 2C). For OS, the median was not reached in patients with early post-treatment Eo elevation, whereas it was 32.4 months (95% CI: 4.2–60.6) in those without early post-treatment Eo elevation (*p* = 0.101; Figure 2D).

**Figure 3:**
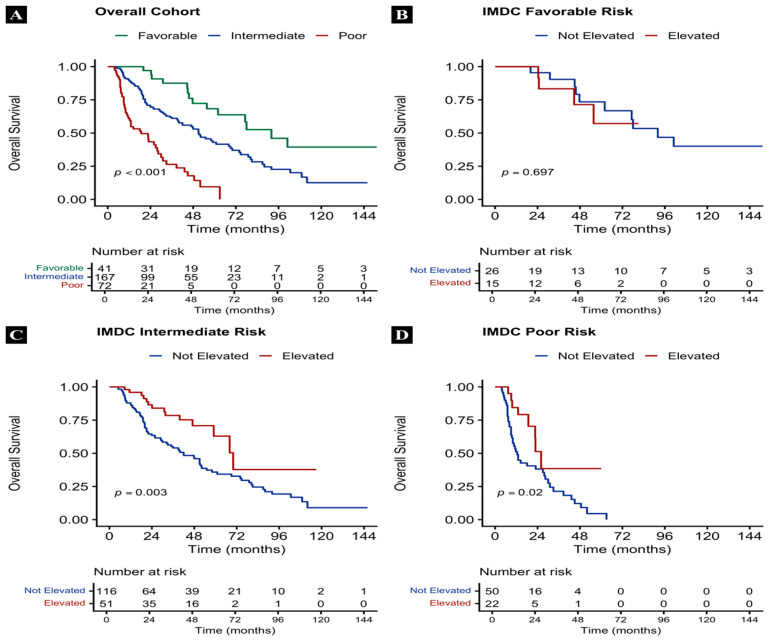
Kaplan–Meier curves for OS according to IMDC risk category in the overall cohort (**A**) and according to early post-treatment Eo elevation status (>5% vs. ≤5%) within the favorable-risk (**B**), intermediate-risk (**C**), and poor-risk (**D**) IMDC subgroups. Abbreviations: Eo: eosinophil; IMDC: International Metastatic Renal Cell Carcinoma Database Consortium; OS: overall survival.

In the overall cohort, OS differed significantly according to IMDC risk category. Median OS was 92.0 months (95% CI: 64.8–119.2) in favorable-risk patients, 51.0 months (95% CI: 39.1–62.9) in intermediate-risk patients, and 18.8 months (95% CI: 10.8–26.8) in poor-risk patients (*p* < 0.001; Figure 3A). When OS was evaluated according to early post-treatment Eo elevation within each IMDC risk category, no significant survival difference was observed in the favorable-risk group. Among favorable-risk patients, median OS was not reached in patients with early post-treatment Eo elevation, whereas it was 92.0 months (95% CI: 63.0–121.0) in those without early post-treatment Eo elevation (*p* = 0.697; Figure 3B). In the intermediate-risk group, patients with early post-treatment Eo elevation had a longer median OS than those without early post-treatment Eo elevation (70.0 months, 95% CI: 57.1–82.9 vs. 42.0 months, 95% CI: 29.9–54.1; *p* = 0.003; Figure 3C). Similarly, in the poor-risk group, median OS was longer in patients with early post-treatment Eo elevation than in those without early post-treatment Eo elevation (26.0 months, 95% CI: 21.6–30.4 vs. 12.5 months, 95% CI: 9.4–15.5; *p* = 0.020; Figure 3D).

### 3.3. Cox Regression Analyses for PFS and OS

The associations between clinicopathologic variables and PFS are presented in Table 3. In univariable Cox regression analysis, intermediate IMDC risk (HR: 1.59, 95% CI: 1.08–2.34, *p* = 0.017) and poor IMDC risk (HR: 3.35, 95% CI: 2.17–5.19, *p* < 0.001) were associated with shorter PFS compared with favorable IMDC risk. Early post-treatment Eo elevation was associated with improved PFS (HR: 0.57, 95% CI: 0.43–0.77, *p* < 0.001). In the multivariable Cox model, early post-treatment Eo elevation remained independently associated with improved PFS (HR: 0.60, 95% CI: 0.44–0.81, *p* = 0.001). IMDC risk category also retained its prognostic significance, with intermediate-risk (HR: 1.62, 95% CI: 1.10–2.40, *p* = 0.014) and poor-risk disease (HR: 3.58, 95% CI: 2.27–5.64, *p* < 0.001) independently associated with shorter PFS. Adrenal metastasis showed a nonsignificant trend toward improved PFS (HR: 0.63, 95% CI: 0.37–1.08, *p* = 0.095), whereas sex, lung metastasis, bone metastasis, and first-line VEGFR-TKI type were not independently associated with PFS.

In the adjusted PFS sensitivity model, early post-treatment Eo elevation was replaced by early post-treatment Eo percentage, analyzed as a continuous variable. A higher early post-treatment Eo percentage remained significantly associated with improved PFS after adjustment for first-line VEGFR-TKI type, sex, IMDC risk category, and lung, bone, and adrenal metastases (HR per 1-percentage-point increase: 0.85, 95% CI: 0.78–0.93, *p* < 0.001).

As shown in Table 4, univariable Cox regression analysis for OS demonstrated that tumor grade III–IV (HR: 1.61, 95% CI: 1.11–2.32, *p* = 0.011), liver metastasis (HR: 1.54, 95% CI: 1.06–2.22, *p* = 0.021), bone metastasis (HR: 1.46, 95% CI: 1.04–2.06, *p* = 0.027), brain metastasis (HR: 2.01, 95% CI: 1.16–3.50, *p* = 0.013), and IMDC risk category were associated with inferior OS. Compared with favorable-risk patients, intermediate-risk patients had an increased risk of death (HR: 2.37, 95% CI: 1.34–4.18, *p* = 0.003), whereas poor-risk patients had a markedly higher mortality risk (HR: 7.27, 95% CI: 3.91–13.51, *p* < 0.001). Sunitinib use was also associated with inferior OS compared with pazopanib (HR: 1.41, 95% CI: 1.01–1.97, *p* = 0.040). However, early post-treatment Eo elevation was associated with improved OS in univariable analysis (HR: 0.46, 95% CI: 0.30–0.71, *p* < 0.001).

In the multivariable Cox model, early post-treatment Eo elevation remained independently associated with favorable OS after adjustment for histology, tumor grade, metastatic sites, IMDC risk category, and first-line VEGFR-TKI type (HR: 0.51, 95% CI: 0.33–0.80, *p* = 0.004). IMDC risk category also retained prognostic significance. Compared with favorable-risk patients, intermediate-risk patients had a higher risk of death (HR: 2.46, 95% CI: 1.37–4.40, *p* = 0.002), while poor-risk patients had a markedly increased mortality risk (HR: 7.86, 95% CI: 4.17–14.81, *p* < 0.001). Tumor grade III–IV (HR: 1.56, 95% CI: 1.07–2.28, *p* = 0.020), liver metastasis (HR: 1.86, 95% CI: 1.27–2.73, *p* = 0.001), bone metastasis (HR: 1.43, 95% CI: 1.00–2.04, *p* = 0.047), and brain metastasis (HR: 2.11, 95% CI: 1.18–3.76, *p* = 0.011) were independently associated with worse OS. Lung metastasis was not independently associated with OS after adjustment (HR: 1.35, 95% CI: 0.93–1.95, *p* = 0.108). Among first-line VEGFR-TKIs, sunitinib was associated with inferior OS compared with pazopanib (HR: 1.43, 95% CI: 1.01–2.01, *p* = 0.038), whereas cabozantinib was not significantly different from pazopanib (HR: 1.09, 95% CI: 0.61–1.95, *p* = 0.766).

In the adjusted OS sensitivity model, in which early post-treatment Eo elevation was replaced by early post-treatment Eo percentage as a continuous variable, a higher early post-treatment Eo percentage remained significantly associated with improved OS after adjustment for first-line VEGFR-TKI type, histology, tumor grade, IMDC risk category, and lung, liver, bone, adrenal, and brain metastases (HR per 1-percentage-point increase: 0.87, 95% CI: 0.77–0.98, *p* = 0.017).

To address the potential guarantee-time bias related to the post-baseline assessment of Eo status, a landmark sensitivity analysis was performed. In the 3-month landmark analysis, early post-treatment Eo elevation remained independently associated with improved OS (adjusted HR: 0.42, 95% CI: 0.24–0.76, *p* = 0.004) and PFS (adjusted HR: 0.55, 95% CI: 0.37–0.82, *p* = 0.004), with consistent results across the 2- and 4-month sensitivity landmarks. In addition, to address the imbalance in first-line VEGFR-TKI distribution between groups, stratified univariable Cox sensitivity analyses were performed according to first-line VEGFR-TKI type. These analyses showed a generally favorable association between early post-treatment Eo elevation and OS. In the pazopanib subgroup, early post-treatment Eo elevation was associated with improved OS (HR: 0.47, 95% CI: 0.26–0.85, *p* = 0.012). In the sunitinib subgroup, a similar favorable trend was observed (HR: 0.51, 95% CI: 0.25–1.03, *p* = 0.060), whereas in the cabozantinib subgroup, the association was not statistically significant (HR: 0.60, 95% CI: 0.13–2.74, *p* = 0.514).

### 3.4. Prognostic Model Performance and Subgroup Sensitivity Analyses

**Figure 4:**
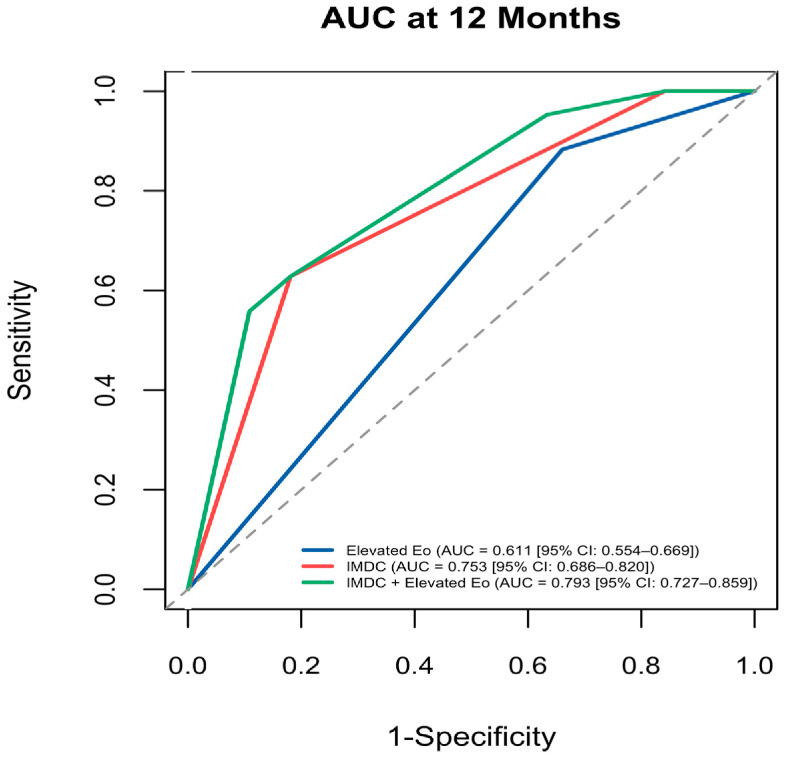
Time-dependent ROC curves assessing the prognostic performance of early post-treatment Eo elevation status (>5% vs. ≤5%), IMDC risk, and the combined IMDC plus Eo elevation model for 12-month OS. Abbreviations: AUC: area under the curve; CI: confidence interval; Eo: eosinophil; IMDC: International Metastatic Renal Cell Carcinoma Database Consortium; OS: overall survival; ROC: receiver operating characteristic.

**Figure 5:**
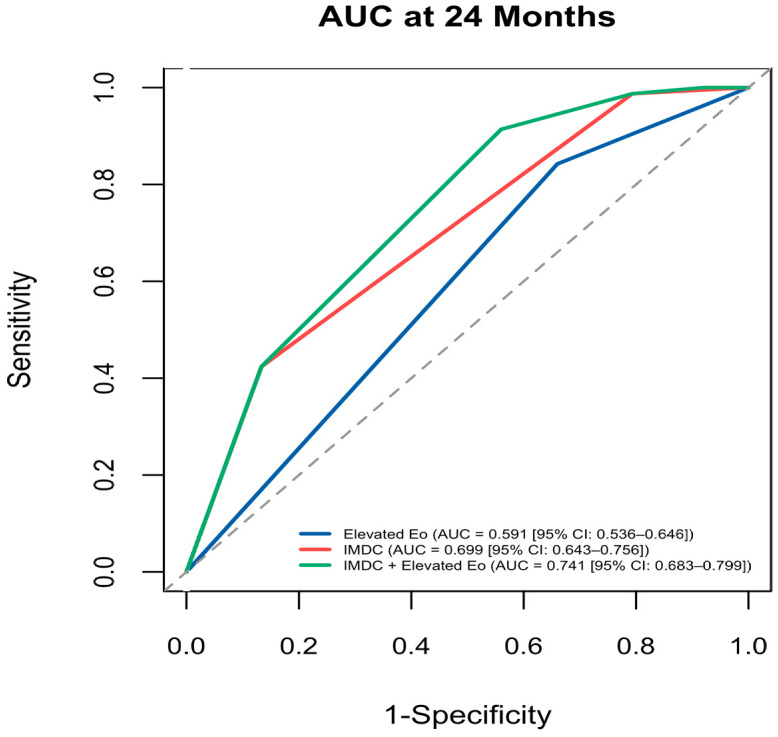
Time-dependent ROC curves assessing the prognostic performance of early post-treatment Eo elevation status (>5% vs. ≤5%), IMDC risk, and the combined IMDC plus Eo elevation model for 24-month OS. Abbreviations: AUC: area under the curve; CI: confidence interval; Eo: eosinophil; IMDC: International Metastatic Renal Cell Carcinoma Database Consortium; OS: overall survival; ROC: receiver operating characteristic.

Time-dependent ROC analyses were performed to evaluate the prognostic performance of early post-treatment Eo elevation, IMDC risk category, and the combined IMDC plus Eo elevation model for OS at 12 and 24 months. As shown in Figure 4, at 12 months, early post-treatment Eo elevation alone showed an AUC of 0.611 (95% CI: 0.554–0.669), whereas the IMDC model had an AUC of 0.753 (95% CI: 0.686–0.820). The combined IMDC plus Eo elevation model demonstrated the highest prognostic performance at 12 months, with an AUC of 0.793 (95% CI: 0.727–0.859). As shown in Figure 5, at 24 months, early post-treatment Eo elevation alone showed an AUC of 0.591 (95% CI: 0.536–0.646), whereas the IMDC model had an AUC of 0.699 (95% CI: 0.643–0.756). The combined IMDC plus Eo elevation model again showed the highest prognostic performance, with an AUC of 0.741 (95% CI: 0.683–0.799).

As shown in Table 5, an adjusted interaction analysis was performed to assess whether the association between early post-treatment Eo elevation and OS differed according to IMDC risk category. IMDC risk was dichotomized as favorable/intermediate versus poor risk, and the model was adjusted for first-line VEGFR-TKI type, histology, tumor grade, and lung, liver, bone, adrenal, and brain metastases. Among patients with favorable/intermediate IMDC risk, early post-treatment Eo elevation was associated with improved OS compared with no Eo elevation (HR: 0.53, 95% CI: 0.31–0.89, *p* = 0.016). Among patients without early post-treatment Eo elevation, poor IMDC risk was strongly associated with inferior OS compared with favorable/intermediate risk (HR: 3.92, 95% CI: 2.61–5.90, *p* < 0.001). Tumor grade III–IV (HR: 1.56, 95% CI: 1.07–2.28, *p* = 0.021), liver metastasis (HR: 1.97, 95% CI: 1.34–2.90, *p* < 0.001), and brain metastasis (HR: 2.52, 95% CI: 1.43–4.44, *p* = 0.001) were independently associated with inferior OS. Bone metastasis showed a borderline association with worse OS (HR: 1.39, 95% CI: 0.98–1.98, *p* = 0.068), whereas lung and adrenal metastases, histology, and first-line VEGFR-TKI type were not significantly associated with OS. The interaction between early post-treatment Eo elevation and poor IMDC risk was not statistically significant (HR: 0.78, 95% CI: 0.31–1.97, *p* = 0.593).

**Figure 6:**
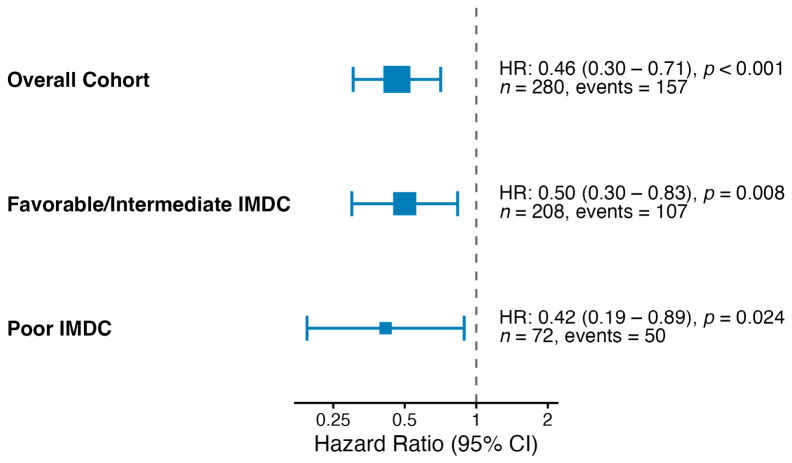
Forest plot of univariable Cox analyses showing the association between early post-treatment Eo elevation status (>5% vs. ≤5%) and OS in the overall cohort and in the dichotomized IMDC risk groups. Abbreviations: CI: confidence interval; Eo: eosinophil; HR: hazard ratio; IMDC: International Metastatic Renal Cell Carcinoma Database Consortium; OS: overall survival.

As shown in Figure 6, univariable Cox subgroup sensitivity analyses were performed according to dichotomized IMDC risk status. In the overall cohort, early post-treatment Eo elevation was associated with improved OS (HR: 0.46, 95% CI: 0.30–0.71, *p* < 0.001). This association remained significant in patients with favorable/intermediate IMDC risk (HR: 0.50, 95% CI: 0.30–0.83, *p* = 0.008) and in those with poor IMDC risk (HR: 0.42, 95% CI: 0.19–0.89, *p* = 0.024).

## 4. Discussion

The present study indicates that early post-treatment Eo elevation during VEGFR-TKI therapy may serve as a clinically meaningful signal of favorable prognosis in mRCC. This association appears to extend beyond conventional baseline risk factors, suggesting that early on-treatment changes in peripheral blood parameters may provide information that is not captured by pretreatment clinicopathologic assessment alone. In this context, early post-treatment Eo elevation may represent a simple dynamic marker reflecting treatment-related host response and disease biology. Its added prognostic contribution to the IMDC model further supports the potential value of integrating early laboratory changes with established risk stratification in routine clinical practice.

Eos have emerged as key participants in the TME, where their actions can either suppress or promote tumor growth depending on the context. They mechanistically contribute to the regulation of tumor immunity by releasing cytotoxic mediators—such as major basic protein and eosinophil cationic protein—that possess direct oncolytic potential [22]. Furthermore, they release chemokines and pro-inflammatory cytokines that aid in the recruitment of T-cells and dendritic cells, enhancing adaptive immune responses [15]. Eos have also been shown to modulate angiogenesis by releasing vascular endothelial growth factors and matrix metalloproteinases [22,23], indicating a mechanistic role in the remodeling of tumor vasculature and influencing drug delivery. These mechanistic findings may partly explain the association between treatment-related early post-treatment Eo elevation and favorable outcomes in cancers, including RCC. Early post-treatment Eo elevation may reflect a favorable immunologic milieu that enhances the anti-angiogenic and immunomodulatory effects of VEGFR-TKIs. Conversely, because of their functional plasticity, Eos may shift from their antitumor activity to supporting angiogenesis, immune suppression, and eventually tumor progression in specific contexts, such as chronic inflammation or tissue remodeling [24]. However, the clinical implications of increased peripheral Eo levels may not be uniformly favorable. Tasaki et al. reported that higher peripheral Eo levels were associated with severe immune-related adverse events in patients with mRCC receiving combined ipilimumab and nivolumab therapy [25]. In RCC specifically, evidence remains limited, but preliminary studies suggest that early post-treatment Eo elevation during systemic therapy is correlated with survival outcomes, consistent with the hypothesis that Eos act as dynamic mediators of antitumor immunity in this disease [21,26,27].

Treatment-period Eo elevation has been recognized as a potential prognostic biomarker across several malignancies, including melanoma, non–small cell lung cancer (NSCLC), and gastrointestinal cancers. Moreira et al. demonstrated that, among patients with melanoma, peripheral Eo elevation occurring at any time during the disease course was associated with higher response rates and significantly longer OS [28]. Alves et al. reported that post-treatment Eo elevation during ICI therapy has also been linked to better treatment response, PFS, and OS for NSCLC patients [29]. A study by McNeel et al. showed that a sipuleucel-T-associated transient rise in Eo counts around week 6 in metastatic castration-resistant prostate cancer was positively correlated with prolonged OS [30]. These findings suggest that treatment-related Eo elevation may reflect early antitumor immune activation and therapeutic benefit in this context.

In RCC, evidence regarding the prognostic role of Eo dynamics remains limited and varies across treatment settings. In patients with mRCC treated with sorafenib, Wang et al. reported that early Eo percentage elevation occurred in approximately one-third of patients and was associated with longer OS [21]. In contrast, in the cytokine-based treatment era, Jeong et al. did not observe a significant difference in Eo increases between responders and non-responders among patients treated with interleukin-2, interferon-α, and 5-fluorouracil [26]. More recently, Herrmann et al. showed that baseline Eo counts and early Eo changes after nivolumab initiation were associated with response and survival outcomes in mRCC, supporting the potential relevance of Eo dynamics in the immunotherapy setting [27]. In the present study, early post-treatment Eo elevation was observed in 31.4% of patients based on the follow-up complete blood count obtained within 15 days before the first radiological assessment, generally scheduled around the third month of VEGFR-TKI therapy. It was independently associated with improved PFS and OS after adjusting for established prognostic factors such as IMDC risk classification and the presence of visceral metastases. Regarding histologic subtypes, our findings suggest that the favorable prognostic impact of early post-treatment Eo elevation appeared more evident in clear-cell RCC, highlighting potential biological differences between clear-cell and non-clear-cell disease subtypes. The present study extends the existing RCC literature by evaluating early post-treatment Eo elevation in patients receiving first-line VEGFR-TKI monotherapy with sunitinib, pazopanib, or cabozantinib, a treatment context in which evidence remains limited. We defined early post-treatment Eo elevation using the follow-up complete blood count obtained within 15 days before the first radiological assessment to align with real-world follow-up patterns, in which complete blood count data may not be consistently available at each monthly visit, and the first radiological response assessment is commonly performed around the third month. Within this clinically relevant window, early post-treatment Eo elevation appeared to provide prognostic information beyond established baseline factors, including IMDC risk classification and metastatic disease characteristics. Moreover, the more evident association in clear-cell RCC suggests that the prognostic relevance of Eo elevation may be influenced by histologic subtype and underlying tumor biology.

Our findings have important clinical implications, providing novel evidence from one of the largest real-world cohorts evaluating early post-treatment Eo elevation during VEGFR-TKI monotherapy. This treatment setting remains relevant for many patients worldwide. Eo percentage is a simple, inexpensive, and non-invasive parameter obtained from routine blood samples. Therefore, assessment of early post-treatment Eo elevation may serve as a readily available marker to refine prognostic evaluation in mRCC, particularly in real-world settings where access to advanced molecular profiling may be limited. Clinically, the >5% threshold may be relevant because it is simple, reproducible, and obtainable from routine complete blood counts without additional cost. However, it should be interpreted as a literature-adapted threshold for early post-treatment Eo elevation rather than a universally established clinical definition of eosinophil increase. Therefore, the optimal timing and cut-off require prospective validation.

Nonetheless, our study has several limitations. First, the retrospective nature of the study may limit generalizability, and while the sample size was relatively robust, the results require validation in well-designed prospective studies. Additionally, the baseline imbalance in metastatic site distribution between the groups should be considered when interpreting the survival findings. Lung metastasis was more common, whereas bone metastasis was less frequent, in patients with early post-treatment Eo elevation. Because the metastatic disease pattern may influence prognosis, clinically relevant metastatic sites, including lung, liver, bone, adrenal, and brain metastases, were included in the multivariable OS model. Early post-treatment Eo elevation remained independently associated with improved OS after this adjustment. Nevertheless, residual confounding related to metastatic site distribution cannot be completely ruled out in a retrospective cohort. Second, Eos were evaluated as percentages rather than absolute counts. Because the Eo percentage may be affected by changes in other leukocyte subsets, it may not accurately reflect the absolute Eo count. Absolute Eo count–based analyses could not be performed because these data were not consistently available across centers. Third, our study was limited to patients treated with VEGFR-TKI monotherapy; therefore, the prognostic role of early post-treatment Eo elevation in the current era of ICI-based combinations remains to be determined. Fourth, the biological mechanisms by which early post-treatment Eo elevation might influence RCC outcomes were not addressed in this study. Prospective and translational studies are needed to establish whether Eos directly contribute to tumor control or act as passive indicators of systemic immune activation. Fifth, although sex-stratified subgroup analyses according to IMDC risk, histology, tumor grade, and metastatic sites would be clinically informative, these analyses were not performed because several resulting strata contained very small numbers of patients and events. Therefore, whether the prognostic association of early post-treatment Eo elevation differs according to sex and detailed clinicopathologic subgroups should be evaluated in larger prospective cohorts. Finally, because early post-treatment Eo elevation was determined from the complete blood count obtained before the first radiological assessment, patients without a pre-imaging blood count—including some with very early progression or death—could not be classified, and the analysis was susceptible to guarantee-time bias. A landmark analysis, with consistent results across sensitivity landmarks, confirmed the primary findings; nonetheless, the requirement for a pre-imaging blood count may have introduced residual selection bias, and the results warrant prospective validation.

## 5. Conclusions

In this study, early post-treatment Eo elevation was associated with favorable survival outcomes in patients with mRCC receiving first-line VEGFR-TKI monotherapy. These findings support early post-treatment Eo elevation as a simple, accessible, and treatment-period prognostic marker that may complement established baseline risk models such as the IMDC classification. Rather than serving as a substitute for established prognostic models, Eo assessment may offer an additional on-treatment perspective for refining early risk stratification. Future prospective studies should validate these findings using standardized Eo assessment intervals. Such studies should define the optimal timing and cut-off for early post-treatment Eo elevation and determine whether its prognostic value applies to different VEGFR-TKI agents, histologic subtypes, and ICI-based combinations.

## Figures and Tables

**Table 1:** Patient characteristics of the overall cohort and according to early post-treatment Eo elevation status.

Characteristics	All Patients (*n* = 280)	No Eo Elevation Group (*n* = 192)	Eo Elevation Group (*n* = 88)	*p* Value
Age, years, median (IQR)	61 (54–67)	60 (53–68)	61 (56–66)	0.361
Sex				0.901
Female	75 (26.8%)	51 (26.6%)	24 (27.3%)	
Male	205 (73.2%)	141 (73.4%)	64 (72.7%)	
Histology				0.054
Clear cell	219 (78.2%)	144 (75%)	75 (85.2%)	
Non-clear cell	61 (21.8%)	48 (25%)	13 (14.8%)	
Tumor grade				0.314
I–II	81 (28.9%)	52 (27.1%)	29 (33%)	
III–IV	199 (71.1%)	140 (72.9%)	59 (67%)	
Metastatic sites				
Lung	201 (71.8%)	129 (67.2%)	72 (81.8%)	0.012
Liver	59 (21.1%)	39 (20.3%)	20 (22.7%)	0.646
Bone	73 (26.1%)	57 (29.7%)	16 (18.2%)	0.042
Adrenal	24 (8.6%)	15 (7.8%)	9 (10.2%)	0.503
Brain	18 (6.4%)	14 (7.3%)	4 (4.5%)	0.384
IMDC				0.744
Favorable	41 (14.6%)	26 (13.5%)	15 (17%)	
Intermediate	167 (59.6%)	116 (60.4%)	51 (58%)	
Poor	72 (25.7%)	50 (26%)	22 (25%)	
Baseline Eo, %, mean (SD)	2.90 (1.00)	2.52 (0.91)	3.73 (0.64)	<0.001
Early post-treatment Eo, %, mean (SD)	3.62 (1.51)	2.77 (0.93)	5.50 (0.45)	

Abbreviations: Eo: eosinophil; IMDC: International Metastatic Renal Cell Carcinoma Database Consortium; SD: standard deviation; IQR: interquartile range.

**Table 2:** Treatment patterns according to early post-treatment Eo elevation status.

Treatment Variable	All Patients	No Eo Elevation Group	Eo Elevation Group	*p* Value
First-line treatment (*n* = 280)				<0.001
Pazopanib	149 (53.2%)	88 (45.8%)	61 (69.3%)	
Sunitinib	101 (36.1%)	82 (42.7%)	19 (21.6%)	
Cabozantinib	30 (10.7%)	22 (11.5%)	8 (9.1%)	
Second-line treatment (*n* = 209)				0.399
Nivolumab	114 (54.5%)	77 (51.3%)	37 (62.7%)	
Axitinib	48 (23%)	35 (23.3%)	13 (22%)	
Everolimus	42 (20.1%)	34 (22.7%)	8 (13.6%)	
Cabozantinib	5 (2.4%)	4 (2.7%)	1 (1.7%)	
Third-line treatment (*n* = 80)				0.550
Nivolumab	25 (31.2%)	17 (30.4%)	8 (33.3%)	
Axitinib	44 (55%)	33 (58.9%)	11 (45.8%)	
Everolimus	8 (10%)	4 (7.1%)	4 (16.7%)	
Cabozantinib	3 (3.8%)	2 (3.6%)	1 (4.2%)	

Abbreviations: Eo: eosinophil.

**Table 3:** Univariable and multivariable Cox analyses investigating the associations between potential prognostic variables and PFS.

Variable	Univariable		Multivariable	
	HR (95% CI)	*p* Value	HR (95% CI)	*p* Value
Age, years	0.99 (0.98–1.01)	0.919		
Sex (male vs. female)	0.81 (0.61–1.08)	0.164	0.95 (0.71–1.28)	0.778
Histology (clear vs. non-clear)	1.06 (0.77–1.45)	0.690		
Tumor grade (III–IV vs. I–II)	1.19 (0.89–1.60)	0.220		
Lung metastasis (present vs. absent)	0.82 (0.62–1.10)	0.199	0.91 (0.67–1.22)	0.536
Liver metastasis (present vs. absent)	1.19 (0.86–1.63)	0.282		
Bone metastasis (present vs. absent)	1.24 (0.93–1.66)	0.132	1.06 (0.78–1.42)	0.700
Adrenal metastasis (present vs. absent)	0.59 (0.35–1.00)	0.050	0.63 (0.37–1.08)	0.095
Brain metastasis (present vs. absent)	1.30 (0.79–2.15)	0.300		
IMDC risk scoring system		<0.001		<0.001
Favorable	1 (reference)		1 (reference)	
Intermediate	1.59 (1.08–2.34)	0.017	1.62 (1.10–2.40)	0.014
Poor	3.35 (2.17–5.19)	<0.001	3.58 (2.27–5.64)	<0.001
First-line VEGFR-TKI		0.004		0.202
Pazopanib	1 (reference)		1 (reference)	
Sunitinib	1.31 (1.00–1.73)	0.048	1.24 (0.93–1.66)	0.127
Cabozantinib	2.02 (1.29–3.16)	0.002	1.36 (0.85–2.19)	0.195
Baseline Eo level, %	0.91 (0.80–1.04)	0.208		
Baseline Eo elevation (>5% vs. ≤5%)	0.90 (0.55–1.48)	0.695		
Early post-treatment Eo level, % ^†^	0.86 (0.79–0.94)	<0.001	0.85 (0.78–0.93)	<0.001
Early post-treatment Eo elevation (>5% vs. ≤5%)	0.57 (0.43–0.77)	<0.001	0.60 (0.44–0.81)	0.001

Abbreviations: CI: confidence interval; Eo: eosinophil; HR: hazard ratio; IMDC: International Metastatic Renal Cell Carcinoma Database Consortium; PFS: progression-free survival; VEGFR-TKI: vascular endothelial growth factor receptor tyrosine kinase inhibitor. ^†^ Early post-treatment Eo percentage was evaluated in a separate multivariable sensitivity model, replacing the dichotomous early post-treatment Eo elevation variable; the two Eo variables were not entered into the same model. This sensitivity model was adjusted for sex, lung, bone, and adrenal metastases, IMDC risk category, and first-line VEGFR-TKI type.

**Table 4:** Univariable and multivariable Cox analyses investigating the associations between potential prognostic variables and OS.

Variable	Univariable		Multivariable	
	HR (95% CI)	*p* Value	HR (95% CI)	*p* Value
Age, years	1.00 (0.98–1.01)	0.692		
Sex (male vs. female)	0.94 (0.66–1.33)	0.736		
Histology (clear vs. non-clear)	1.30 (0.90–1.88)	0.162	1.08 (0.72–1.62)	0.694
Tumor grade (III–IV vs. I–II)	1.61 (1.11–2.32)	0.011	1.56 (1.07–2.28)	0.020
Lung metastasis (present vs. absent)	1.19 (0.83–1.70)	0.338	1.35 (0.93–1.95)	0.108
Liver metastasis (present vs. absent)	1.54 (1.06–2.22)	0.021	1.86 (1.27–2.73)	0.001
Bone metastasis (present vs. absent)	1.46 (1.04–2.06)	0.027	1.43 (1.00–2.04)	0.047
Adrenal metastasis (present vs. absent)	0.55 (0.25–1.18)	0.125	0.64 (0.29–1.37)	0.254
Brain metastasis (present vs. absent)	2.01 (1.16–3.50)	0.013	2.11 (1.18–3.76)	0.011
IMDC risk scoring system		<0.001		<0.001
Favorable	1 (reference)		1 (reference)	
Intermediate	2.37 (1.34–4.18)	0.003	2.46 (1.37–4.40)	0.002
Poor	7.27 (3.91–13.51)	<0.001	7.86 (4.17–14.81)	<0.001
First-line VEGFR-TKI		0.083		0.110
Pazopanib	1 (reference)		1 (reference)	
Sunitinib	1.41 (1.01–1.97)	0.040	1.43 (1.01–2.01)	0.038
Cabozantinib	1.50 (0.87–2.59)	0.143	1.09 (0.61–1.95)	0.766
Baseline Eo level, %	0.93 (0.79–1.08)	0.360		
Baseline Eo elevation (>5% vs. ≤5%)	0.86 (0.46–1.59)	0.635		
Early post-treatment Eo level, % ^†^	0.88 (0.79–0.98)	0.024	0.87 (0.77–0.98)	0.017
Early post-treatment Eo elevation (>5% vs. ≤5%)	0.46 (0.30–0.71)	<0.001	0.51 (0.33–0.80)	0.004

Abbreviations: CI: confidence interval; Eo: eosinophil; HR: hazard ratio; IMDC: International Metastatic Renal Cell Carcinoma Database Consortium; OS: overall survival; VEGFR-TKI: vascular endothelial growth factor receptor tyrosine kinase inhibitor. ^†^ Early post-treatment Eo percentage was evaluated in a separate multivariable sensitivity model, replacing the dichotomous early post-treatment Eo elevation variable; the two Eo variables were not entered into the same model. This sensitivity model was adjusted for first-line VEGFR-TKI type, histology, tumor grade, IMDC risk category, and lung, liver, bone, adrenal, and brain metastases.

**Table 5:** Adjusted interaction analysis for OS according to early post-treatment Eo elevation and dichotomized IMDC risk category.

Variable	HR	95% CI		*p* Value
		Lower	Upper	
Histology (clear-cell vs. non-clear-cell)	1.08	0.73	1.60	0.703
Tumor grade (III–IV vs. I–II)	1.56	1.07	2.28	0.021
Lung metastasis, present vs. absent	1.37	0.95	1.97	0.090
Liver metastasis, present vs. absent	1.97	1.34	2.90	<0.001
Bone metastasis, present vs. absent	1.39	0.98	1.98	0.068
Adrenal metastasis, present vs. absent	0.67	0.31	1.43	0.299
Brain metastasis, present vs. absent	2.52	1.43	4.44	0.001
Poor IMDC risk vs. favorable/intermediate risk	3.92	2.61	5.90	<0.001
Early post-treatment Eo elevation (>5% vs. ≤5%)	0.53	0.31	0.89	0.016
Early post-treatment Eo elevation × poor IMDC risk	0.78	0.31	1.97	0.593
First-line VEGFR-TKI				0.147
Pazopanib	1 (reference)			
Sunitinib	1.39	0.99	1.95	0.056
Cabozantinib	1.05	0.59	1.85	0.869

Abbreviations: CI: confidence interval; Eo: eosinophil; HR: hazard ratio; IMDC: International Metastatic Renal Cell Carcinoma Database Consortium; OS: overall survival; VEGFR-TKI: vascular endothelial growth factor receptor tyrosine kinase inhibitor.

## Data Availability

Data are available upon reasonable request. The data are not publicly available due to patient privacy and ethical considerations.

## Abbreviations

AUC	area under the curve
CI	confidence interval
Eo	eosinophil
HR	hazard ratio
ICI	immune-checkpoint inhibitor
IMDC	International Metastatic Renal Cell Carcinoma Database Consortium
IQR	interquartile range
mRCC	metastatic renal cell carcinoma
NSCLC	non–small cell lung cancer
PFS	progression-free survival
RCC	renal cell carcinoma
ROC	receiver operating characteristic
OS	overall survival
SD	standard deviation
TME	tumor microenvironment
TKI	tyrosine kinase inhibitor
VEGFR	vascular endothelial growth factor receptor
VEGFR-TKI	vascular endothelial growth factor receptor tyrosine kinase inhibitor
